# “Interstitial lung abnormalities”: translation to and use in Portuguese

**DOI:** 10.1590/0100-3984.2023.0114-en

**Published:** 2024-04-15

**Authors:** Tassia Regina Yamanari, Ricardo Valarelli Auad, Alexandre Dias Mançano, Marcel Koenigkam-Santos, Pablo Rydz Pinheiro Santana, Arthur Soares Souza Júnior, Rodrigo Caruso Chate, Marcio Valente Yamada Sawamura

**Affiliations:** 1 Instituto de Radiologia do Hospital das Clínicas da Faculdade de Medicina da Universidade de São Paulo (InRad/HC-FMUSP), São Paulo, SP, Brazil; 2 Hospital Sírio-Libanês, São Paulo, SP, Brazil; 3 Hospital do Coração, São Paulo, SP, Brazil; 4 Hospital Sírio-Libanês Brasília, Brasília, DF, Brazil; 5 Universidade de São Paulo (USP), Campus de Bauru, Bauru, SP, Brazil; 6 University of Florida, Jacksonville, FL, USA.; 7 7. Ultra-X - Medicina Diagnóstica, São José do Rio Preto, SP, Brazil; 8 Instituto do Coração do Hospital das Clínicas da Faculdade de Medicina da Universidade de São Paulo (InCor/HC-FMUSP), São Paulo, SP, Brazil; 9 Hospital Israelita Albert Einstein, São Paulo, SP, Brazil

**Keywords:** Lung diseases, interstitial, Tomography, X-ray computed, Pulmonary fibrosis, Incidental findings, Doenças pulmonares intersticiais, Tomografia computadorizada, Fibrose pulmonar, Achados incidentais

## Abstract

**Objective:**

To conduct a survey on the use of the term “interstitial lung abnormalities” in radiology reports in Brazil, propose an appropriate Portuguese-language translation for the term, and provide a brief review of the literature on the topic.

**Materials and Methods:**

A survey was sent via electronic message to various radiologists in Brazil, asking about their familiarity with the term, which translation of the term they use in Portuguese, and whether they use the criteria proposed by the Fleischner Society.

**Results:**

A total of 163 responses were received, from all regions of Brazil. Although the vast majority (88%) of the respondents stated that they were familiar with the term “interstitial lung abnormalities”, there was considerable variation regarding the equivalent term they used in Portuguese.

**Conclusion:**

We suggest that the term “anormalidades pulmonares intersticiais” be used in order to standardize radiology reports and disseminate knowledge of these findings in Brazil.

## Abstract (pt)

**Objetivo:**

Fazer um levantamento sobre o uso do termo *interstitial lung abnormalities* nos laudos radiológicos no Brasil, propor uma tradução para o termo e fazer uma breve revisão sobre o tema.

**Materiais e Métodos:**

Foi enviada uma pesquisa, por meio de mensagem eletrônica, para diversos radiologistas de todo o Brasil, questionando sobre a familiarização com o termo, qual tradução em português utilizam e se usam os critérios propostos pela diretriz da Sociedade Fleischner.

**Resultados:**

Foram recebidas 163 respostas de todas as regiões do Brasil e a grande maioria dos radiologistas respondeu estar familiarizado com o termo *interstitial lung abnormalities* (88%), mas houve grande variação em relação ao termo utilizado como tradução para o português.

**Conclusão:**

Sugerimos a padronização do termo “anormalidades pulmonares intersticiais”, a fim de uniformizar os relatórios radiológicos e difundir esta entidade no País.

## INTRODUCTION

The term “interstitial lung abnormalities” (ILAs) can be most directly translated to Portuguese as *anormalidades pulmonares intersticiais*. This set of findings is characterized by abnormalities consistent with interstitial lung disease (ILD) on computed tomography (CT) in patients with no clinical suspicion of the disease^(1)^. It has gained relevance in recent years, after a number of scientific studies showed that such abnormalities can represent an initial stage of pulmonary fibrosis and that there is progression of those findings in some patients^(2-4)^. That culminated in the publication of a position paper by the Fleischner Society, standardizing the definition of these findings, in 2020^(1)^. In Brazil, however, we observed that this term is translated differently by different radiologists, which can create confusion in their understanding and case management.

The objective of this study was to conduct a survey regarding the use of this term in radiology reports in Brazil. We also provide a brief review of the literature on the topic.

## MATERIALS AND METHODS

Between September 1 and September 12 of 2023, a survey was sent via electronic message to several radiologists, throughout Brazil, who are considered references in thoracic radiology because of their previous participation in lectures, national conferences, and scientific publications. Each radiologist was also asked to share the survey with their coworkers and residents.

The survey consisted of questions regarding the length of experience in radiology, the Brazilian state in which they work, whether they were familiar with the term ILAs, which translation of the term they use, and whether they used the criteria proposed by the Fleischner Society^(1)^. Permission to use the data anonymously for academic purposes was also requested.

## RESULTS

A total of 163 responses/permissions were received, from all five regions of Brazil: 103 (63.1%) from the southeastern region; 32 (19.6%) from the northeastern region; 17 (10.4%) from the central-west region; nine (5.5%) from the southern region; and two (1.2%) from the northern region. The vast majority of the responses (60%) were from the state of São Paulo.

Of the 163 respondents, 24 (14.7%) were residents or interns. Of the 139 radiologists, 64 (39.2%) had up to five years of experience, 33 (20.2%) had 6-10 years of experience, 28 (17.1%) had 11-20 years of experience, and 14 (8.5%) had more than 20 years of experience.

The vast majority (88%) of the respondents stated that they were familiar with the term ILAs. However, only 60% reported using the diagnostic and classification criteria recommended by the Fleischner Society^(1)^.

Regarding the term used in Portuguese as a translation of ILAs, there was considerable variation among the respondents. The most common term was *alterações pulmonares intersticiais* (“interstitial lung changes”, used by 41.7%), followed by *alterações pulmonares incipientes* and *anormalidades pulmonares intersticiais* (“incipient lung changes” and “ILAs”, each used by 20.2%); *pneumopatia intersticial incipiente* (“incipient ILD”, used by 11.0%); *alterações intersticiais pulmonares incidentais* (“incidental pulmonary interstitial changes”, used by 3.0%); *anormalidades intersticiais incipientes* (“incipient interstitial abnormalities”, used by 1.2%); *anormalidades intersticiais incidentais* (“incidental interstitial abnormalities”, used by 0.6%); *anormalidades pulmonares intersticiais incipientes* (“incipient ILAs”, used by 0.6%); *sinais de intersticiopatia incipiente* (“signs of incipient interstitial disease”, used by 0.6%); and *intersticiopatia* (“interstitial disease”, used by 0.6%).

## DISCUSSION

Typically detected as incidental findings on CT of the chest, ILAs pose a risk of progression to ILD. The prevalence of ILAs is estimated to be approximately 10% in the general population, increasing with age and smoking history. Other risk factors for ILAs include male gender and exposure to air pollution, as well as occupational exposure to vapors, dust, or smoke. There is an association between ILAs and worse clinical outcomes, a finding of ILAs having a negative impact on patient survival^(1-6)^.

**Figure 1:**
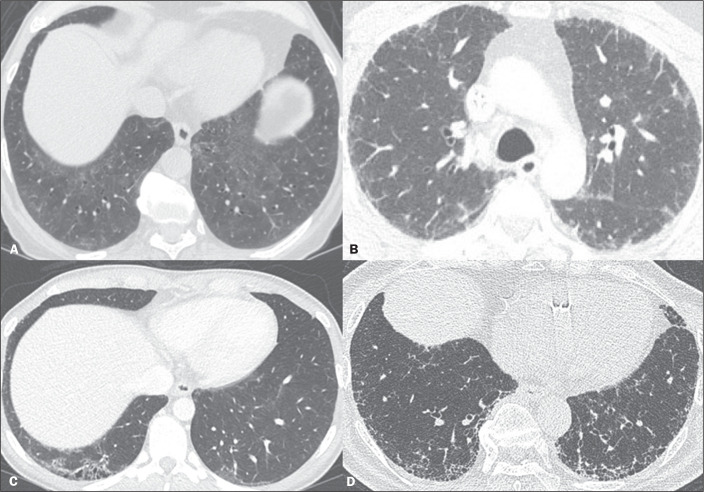
ILA subtypes. A: Non-subpleural ILAs. Reticular and ground-glass opacities in the lung bases. B: Subpleural non-fibrotic ILAs. Subpleural reticular and ground-glass opacities in the lungs, without signs of fibrosis. C,D: Subpleural fibrotic ILAs. Subpleural reticular opacities with traction bronchiolectasis.

**Figure 2:**
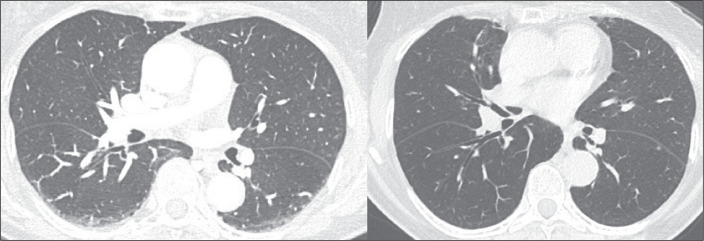
CT findings that should not be considered ILAs. Lung opacities in the posterior portion of the lungs that are position-dependent, resolving when images are acquired in the prone position on the same day.

**Figure 3:**
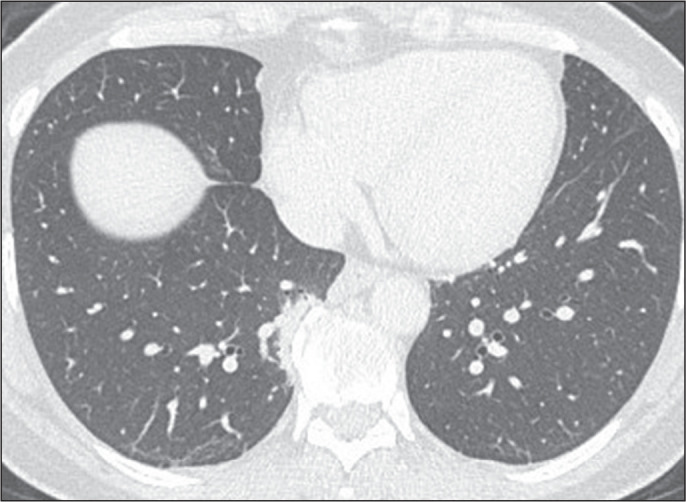
CT findings that should not be considered ILAs. Paraspinal fibrosis in the right lower lung lobe, adjacent to spinal osteophytes.

Because of the clinical importance of identifying ILAs, the Fleischner Society defined standardized descriptive terms, as well as proposing a multidisciplinary management plan for cases in which these changes are found^(1)^. In the Fleischner Society paper, ILAs are defined as non-position-dependent pulmonary opacities, with diffuse distribution, involving at least 5% of any lung zone (the upper, middle and lower lung zones being separated by the levels of the inferior aortic arch and the right inferior pulmonary vein), detected incidentally in individuals without suspected ILD. The aim of establishing a 5% cutoff is to exclude minimal or dubious CT findings. The findings can include ground-glass opacities, reticulated opacities, architectural distortion, bronchiectasis, traction bronchiolectasis, honeycombing, and non-emphysematous cysts (Figure 1). There are some CT findings that should not be considered ILAs, such as position-dependent pulmonary atelectasis (Figure 2), paraspinal fibrosis adjacent to osteophytes (Figure 3), dendriform pulmonary ossification, thickening of the interlobular septum due to interstitial edema, and focal or unilateral opacities (such as those related to bronchial aspiration). In populations at high risk for ILD (such as patients with a family history of ILD, a known diagnosis of connective tissue disease, or significant occupational exposure to an agent known to be associated with ILD), changes identified during CT screening should not be considered ILAs because they are not incidental^(1,2)^. In addition, ILAs can be classified, according to their location(s) and the presence or absence of fibrosis, into three subtypes-non-subpleural, subpleural non-fibrotic, and subpleural fibrotic-fibrosis being characterized by architectural distortion with traction bronchiectasis or bronchiolectasis, with or without honeycombing^(1,2)^.

**Figure 4:**
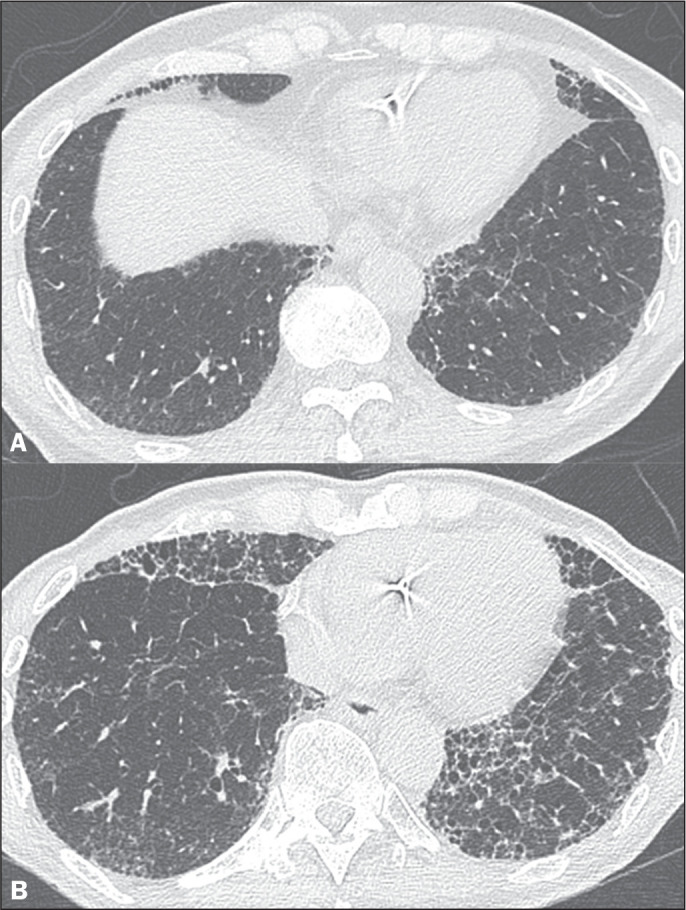
Evolution of subpleural fibrotic ILAs over five years. A: Chest CT showing reticular opacities associated with traction bronchiolectasis in the lung periphery. B: Chest CT acquired five years later, showing progression of the ILAs, with increased fibrosis, together with honeycombing and architectural distortion.

In the present study, 60% of the radiologists surveyed stated that they use the ILA subtypes in their chest CT reports. This information is relevant, because subpleural non-fibrotic and subpleural fibrotic ILAs are considered to present a high risk for progression (Figure 4). The fibrotic subtype is also associated with higher mortality than that observed for individuals without ILAs^(3,7,8)^.

As previously mentioned, the vast majority of the survey respondents stated that they were familiar with ILAs. However, the great variation among them in terms of how they translate the term to Portuguese in their chest CT reports could create confusion in the interpretation of these findings. We believe it is essential to standardize the term in radiology reports in order to improve interdisciplinary communication and case management.

Of the radiologists who responded to the survey, 31.8% stated that they used the terms *alterações pulmonares incipientes* (“incipient lung changes”), *pneumopatia intersticial incipiente* (“incipient ILD”), or *sinais de intersticiopatia incipiente* (“signs of incipient interstitial disease”). In this context, the use of the term “incipient” implies that an alteration is in an initial phase and will presumably progress. However, some studies have shown that not all individuals with ILAs present radiological progression or develop ILD, and that some ILAs even regress^(8)^. Likewise, although the term *alterações pulmonares intersticiais* (“interstitial lung changes”) was routinely used by approximately 40% of the survey respondents, it lacks precision. Despite having similar meanings in the Portuguese language, there is a more pronounced distinction between “abnormality” and “change” in the English language, the latter term being more commonly associated with the idea of replacement or difference from a previous state, making it less appropriate in this context.

Our study has some limitations. The choice of specific radiologists as recipients of the survey represents a clear selection bias. Other potential limitations are the use of electronic media to carry out the survey and the fact that the majority of the respondents had five or fewer years of experience in radiology.

## CONCLUSION

We believe that direct translation of the term ILAs to *anormalidades pulmonares intersticiais* would be the most appropriate for use in Portuguese. We also believe that it would be appropriate to identify the subpleural non-fibrotic and subpleural fibrotic ILA subtypes, with the aim of standardizing radiology reports, thus improving communication between radiologists and professionals in other medical specialties, as well as promoting uniformity in future clinical research. In addition, measures to promote the use of this term in Portuguese are important for raising awareness and consolidating the knowledge of the concept.
